# The first complete mitochondrial genome of *MAMMILLA* from *Mammilla mammata* (Littorinimorpha: Naticidae)

**DOI:** 10.1080/23802359.2019.1698350

**Published:** 2019-12-12

**Authors:** Shengping Zhong, Lianghua Huang, Guoqiang Huang, Yonghong Liu, Weixing Wang

**Affiliations:** a Institute of marine drugs, Guangxi University of Chinese Medicine, Nanning, China;; b Key Laboratory of Marine Biotechnology, Guangxi Institute of Oceanology, Beihai, China;; c School of Artificial Intelligence, The Open University of Guangdong, Guangzhou, China

**Keywords:** Mitochondrial genome, *Mammilla mammata*, Littorinimorpha

## Abstract

*Mammilla mammata* is an ecologically and economically important species of Caenogastropoda, which is the largest and most evolutionary successful group of marine gastropods. However, the phylogenetic relationships between the families and superfamilies within Caenogastropoda have been debated. In this study, we report the first complete mitochondrial genome of *Mammilla* from *M. mammata*. The mitogenome has 15,300 base pairs (71.4% A + T content) and made up of total of 37 genes (13 protein-coding, 22 transfer RNAs and 2 ribosomal RNAs), and a control region. This study was the first available complete mitogenomes of *Mammilla* and will provide useful genetic information for future phylogenetic and taxonomic classification of Naticidae.

The Caenogastropoda is the largest and most evolutionary successful group of marine gastropods, which comprises of about 136 extant and 65 extinct families including a large number of ecologically and commercially important marine families (Colgan et al. 2007). These gastropods are highly diverse in the morphology and have adapted to very different marine environment. Moreover, some gastropods including *Mammilla mammata* are economically important as luxury seafood and valuable ingredients of traditional medicines (Ahmad et al. 2018). However, the phylogenetic relationships between the families and superfamilies within Caenogastropoda remain largely unresolved and monophyly of Littorinimorpha and Neogastropoda has been debated (Osca et al. 2015). The complete mitochondrial genome can be used to reconstruct robust phylogenies for phylogenetic and taxonomy studying, but adequate mitogenome information about the Littorinimorpha is still limited. Here, we report the first complete mitochondrial genome sequence of *Mammilla*, which will provide a better insight into phylogenetic assessment and taxonomic classification.

A tissue samples of *M. mammata* from five individuals were collected from GuangXi province, China (Beihai, 21.450778 N, 109.501951 E), and the whole body specimen (#GR0316) were deposited at Marine biological Herbarium, Guangxi Institute of Oceanology, Beihai, China. The total genomic DNA was extracted from the muscle of the specimens using an SQ Tissue DNA Kit (OMEGA, Guangzhou, China) following the manufacturer’s protocol. DNA libraries (350 bp insert) were constructed with the TruSeq NanoTM kit (Illumina, San Diego, CA) and were sequenced (2 × 150 bp paired-end) using HiSeq platform at BGI Company, China. Mitogenome assembly was performed by MITObim (Hahn et al. 2013). The complete mitogenome of *Naticarius hebraeus* (GenBank accession number: NC_028002) was chosen as the initial reference sequence for MITObim assembly. Gene annotation was performed by MITOS (Bernt et al. 2013).

The complete mitogenome of *M. mammata* was 15,300 bp in length (GenBank accession number: MN596871), and containing the typical set of 13 protein-coding, 22 tRNA and 2 rRNA genes, and a putative control region. The overall base composition of the mitogenome was estimated to be A 31.4%, T 40.0%, C 13.4% and G 15.2%, with a high A + T content of 71.4%, which is similar, but slightly higher than *Hemifusus tuba* (68.2%) (Zhong et al. 2019). The mitogenomic phylogenetic analyses showed that *M. mammata* was first clustered with *Naticarius hebraeus* and *Glossaulax reiniana* within family Naticidae clade with high bootstrap value (Figure 1), and the grade of Littorinimorpha was supported, which is consistent with the phylogenetic analyses within Caenogastropoda based on the deduced amino acid sequences of concatenated mitogenome protein coding genes (Osca et al. 2015). The complete mitochondrial genome sequence of *M. mammata* was the first sequenced mitogenome in *Mammilla*, which will contribute to further phylogenetic and comparative mitogenome studies of Naticidae, and related families.

**Figure 1.**
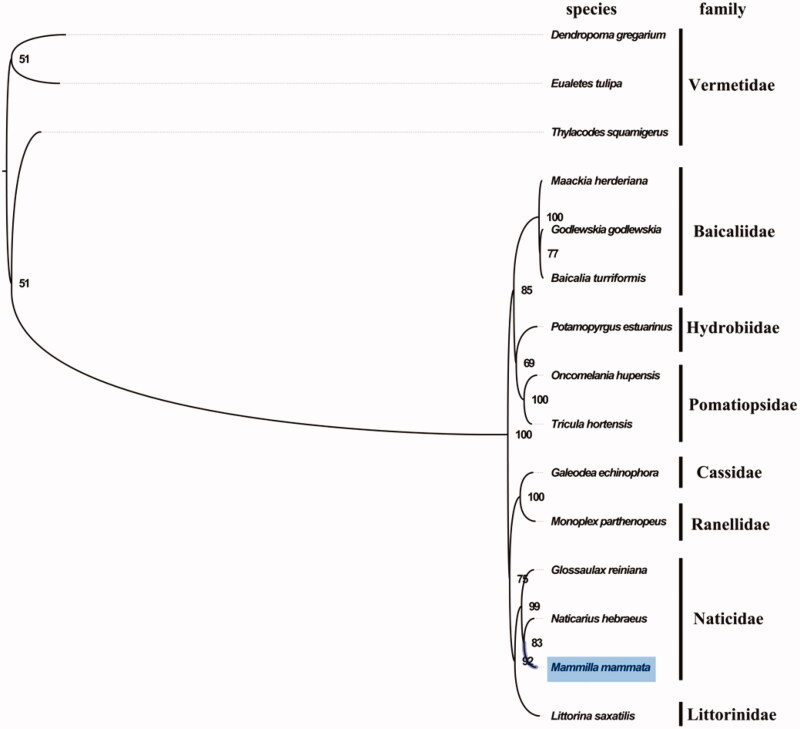
Phylogenetic tree of 15 species in order Littorinimorpha. The complete mitogenomes is downloaded from GenBank and the phylogenic tree is constructed by maximum-likelihood method with 100 bootstrap replicates. The bootstrap values were labeled at each branch nodes. The gene’s accession number for tree construction is listed as follows: *Thylacodes squamigerus* (NC_014588), *Maackia herderiana* (NC_035871), *Godlewskia godlewskia* (NC_035870), *Baicalia turriformis* (NC_035869), *Potamopyrgus estuarinus* (NC_021595), *Oncomelania hupensis* (NC_013073), *Tricula hortensis* (NC_013833), *Galeodea echinophora* (NC_028003), *Monoplex parthenopeus* (NC_013247), *Glossaulax reiniana* (NC_041162), *Naticarius hebraeus* (NC_028002), *Littorina saxatilis* (NC_030595), *Dendropoma gregarium* (NC_014580), and *Eualetes tulipa* (NC_014585).
